# Basic research opportunities focused on bio-based and bio-inspired materials and potential applications

**DOI:** 10.3389/fchem.2014.00024

**Published:** 2014-05-13

**Authors:** Stephanie A. Nick McElhinny, Jennifer J. Becker

**Affiliations:** U.S. Army Research OfficeResearch Triangle Park, NC, USA

**Keywords:** bio-based materials, bio-inspired materials, hybrid materials, biomimetic systems, basic research

The U.S. Army Research Office (ARO) serves as the Army's premier extramural basic research agency in the engineering, physical, information and life sciences. ARO drives the national basic research agenda and programs in these areas to create new scientific discoveries and increase knowledge through high-risk, high-payoff research opportunities with academia and industry. ARO also ensures that the results of these efforts are made available to the Army research and development community for the pursuit of long-term technological applications. ARO is supporting emerging research opportunities in the areas of bio-based and bio-inspired materials that focus on four primary goals: (i) Using biology to produce materials, (ii) Using biology as a material, (iii) Integrating biology with synthetic materials, and (iv) Imparting properties inherent in biological systems to materials.

## Using biology to produce biological, non-biological, and hybrid materials

Biological organisms have evolved complex synthetic capabilities that are often unrivaled, if not impossible, by traditional chemical approaches, with products ranging from small molecule chemicals to biological polymers to macromolecular assemblies with complex catalytic or mechanical activities to metallic and semiconductor nanoparticles. In addition to the many valuable products naturally produced by biological systems, such as the chemotherapeutic agent Taxol produced by an endophytic fungus living within the bark of the Pacific Yew tree, biological systems are also being engineered to produce specific products of interest. From the advent of genetic engineering approaches in the 1970s to the recent emergence of the field of synthetic biology, the research community continues to push the boundaries of biological engineering and the complexity of the products that are synthesized by biological systems.

The capability to efficiently produce valuable chemicals and materials using biological systems is particularly desirable due to the mild synthesis conditions and general lack of toxic byproducts. Biological synthetic platforms are also conducive to the design and production of hybrid products that contain both biological and non-biological elements. Currently supported research is focused on engineering microorganisms to incorporate unnatural amino acids into protein polymer chains. Successful efforts could enable the production of hybrid polymers that contain strategically located biological functional groups to endow materials produced from these polymers with specific recognition or reactive functions. Such strain engineering also opens the possibility to one day produce traditional chemical polymers in a biological system with control over monomer sequence—a fundamental characteristic of biological polymers such as DNA, RNA, and proteins that has remained elusive for non-biological polymers.

The potential applications of biologically produced materials could be vast, depending on the types of materials that can be made and the quantities of these materials that can be reliably produced. Biological production platforms could be optimized to produce naturally occurring products, including therapeutic compounds, alternative fuels or enzymes. Engineered biological systems could produce hybrid products with both biological and non-biological elements, leading to materials with both traditionally desirable properties, such as high strength and stability, as well as biological functionality, such as molecular recognition or enzymatic reactivity. Engineered biological systems also hold potential to produce entirely novel products not yet imagined, with the type of advanced genetic pathway design and assembly envisioned by the DARPA Living Foundries program.

## Using biology as a material

Biological molecules and macromolecular assemblies form intricate and precise architectures at the nano- and micro-scale with nanometer resolution of structural features. This level of precision is not yet accessible with traditional top-down fabrication approaches, providing an opportunity to utilize biological assemblies as materials themselves.

DNA nanotechnology enables the production of precisely designed nanometer and micron scale 2D and 3D structures with complex features including curvature and inner channels and cavities, and recent advances provide novel assembly approaches to “carve” precise 3D structures from a “molecular canvas” of DNA (Ke et al., 2012). As a material, DNA nanostructures could be used as templates to organize functional elements with control over spatial orientation at the nanometer scale or as a “mold” for the production of inorganic materials with nano scale features. A major research program is currently exploring the use of DNA nanostructures as a surrogate for the 3D spatial control of the cellular environment to promote the activity of a biochemical pathway in an *in vitro* setting. DNA nanostructures have also been used to design molecular machines and have potential applications in molecular scale electronics and targeted drug delivery.

Proteins also assemble into organized structures that can be utilized as materials. Perhaps one of the most well-known protein-based materials is the protein fiber. Many different proteins assemble into fibers and fibrils, including collagen, elastin, silk, and amyloid proteins, with each protein fiber exhibiting unique material properties. Amyloid fibrils have been used as a nanoscaffold for enzyme immobilization and stabilization. Glucose oxidase and organophosphate hydrolase have been successfully immobilized onto amyloid fibrils and demonstrated an increase in thermal stability while maintaining activity (Pilkington et al., 2010; Raynes et al., 2011). Proteins also assemble into complex 3D structures, including viral capsids which vary dramatically in size and shape, and have potential to be used as molecular delivery vessels or templates for the ordered display of functional elements. Currently supported research is exploring the Tobacco mosaic virus as a biological building block for engineered systems. This nanotube-shaped virus may be genetically and chemically modified to tailor its physical properties and is compatible with some conventional microfabrication processes (Fan et al., 2013).

## Integrating biology with synthetic materials

An ability to integrate biological elements with synthetic materials may enable systems that marry the specificity and reactivity of biology with the stability and predictability of synthetic material systems. Integrating these two worlds is no simple task, and major research programs are focusing on elucidating key elements that support retention of biological structure and function when these two material classes are merged.

A significant effort is focused on understanding the interactions at the interface between immobilized proteins and a chemical surface, and how the chemical and physical environment at this interface impacts biological structure and function. Future applications that could be realized by scientific advances in this area include reactive coatings, bioactive textile treatments, advanced chemical sensors, anti-biofouling approaches, and catalysis. Another major program aims to integrate biological and biomimetic synthetic cellular elements to create novel artificial cells with unprecedented spatial and temporal control of genetic circuits and biological pathways. These hybrid biological/synthetic cells have the potential to provide a fundamentally new chassis for synthetic biology that addresses the critical challenge of instilling increased control and stability to engineered biological systems.

## Imparting properties inherent in biological systems to materials

For certain applications and use scenarios, a fully synthetic material or chemical system that exhibits properties inherent in biological systems, without the inclusion of biological elements, would be ideal. Living biological systems have many desirable characteristics. They can be dynamic, self-organizing, multi-functional, responsive, and complex. They can autonomously adapt to their changing environment. Novel approaches which impart these properties to non-biological synthetic chemical and material systems could enable significant new capabilities.

One property of biological systems that would provide novel functionality for synthetic material systems is the precise temporal and spatial regulation of activity. Biology has evolved complex mechanisms to tightly regulate molecular functions. This regulation can be achieved via changes in chemical, optical, electrical, and mechanical stimulation of active molecular elements. A major program will aim to understand the molecular mechanisms by which living cells regulate intracellular biochemical activity with mechanical force and to reproduce and analyze these force-activated mechanisms in virtual and synthetic materials. Scientific advances in this area could lead to sense-and-respond systems that incorporate force-activation to maximize multi-modal functionality, reactive coatings, novel sensor paradigms, and self-healing materials.

Major research programs are also investigating methods for imparting multi-functionality and dynamic, responsive behavior into purely synthetic chemical and material systems. Potential applications of these systems include smart sensors, self-healing and self-repairing materials, reconfigurable materials, and controlled release of materials. For example, micelle/nanoparticle composite systems are possible in which different environmental stimuli (pH, temperature, oxidation) result in different material responses (Zhuang et al., 2013). In another example, micelle systems have been made in which the morphology of the micelle can be changed by environmental triggers. An enzymatic reaction or addition/removal of complimentary DNA can cause micelles to alternate between spherical and cylindrical forms resulting in bulk material changes (Randolph et al., 2012).

The revolutionary basic research ARO is supporting in the areas of bio-based and bio-inspired materials as summarized above has the potential to impact diverse applications in sensing, alternative fuels, molecular scale electronics, targeted drug delivery, and autonomously adaptive materials. This research has the potential to harness the power of biology with the control and stability of material and chemical systems to provide revolutionary scientific advances.

### Conflict of interest statement

The authors declare that the research was conducted in the absence of any commercial or financial relationships that could be construed as a potential conflict of interest.
